# Effect of Small Amount of Ni Addition on Microstructure and Fatigue Properties of Sn-Sb-Ag Lead-Free Solder

**DOI:** 10.3390/ma14143799

**Published:** 2021-07-07

**Authors:** Mizuki Yamamoto, Ikuo Shohji, Tatsuya Kobayashi, Kohei Mitsui, Hirohiko Watanabe

**Affiliations:** 1 Graduate School of Science and Technology, Gunma University, Kiryu 3768515, Japan; t201b095@gunma-u.ac.jp (M.Y.); kobayashi.t@gunma-u.ac.jp (T.K.); watanabe-hirohiko@fujielectric.com (H.W.); 2 Production Technology Development Section, Fuji Electric Co., Ltd., Hino 1918502, Japan; mitsui-kouhei@fujielectric.com

**Keywords:** Sn-6.4Sb-3.9Ag, lead-free solder, Ni addition, microstructure, tensile properties, low cycle fatigue properties, EBSD analysis

## Abstract

The effect of the addition volume of Ni on the microstructures and tensile and fatigue properties of Sn-6.4Sb-3.9Ag (mass%) was investigated using micro-size specimens. The addition of Ni into Sn-6.4Sb-3.9Ag tends to increase the number of grains formed in the solidification process and produce a high-angle grain boundary. An amount of 0.1% proof stress of Sn-6.4Sb-3.9Ag decreases with an increase in the Ni addition volume at a strain rate of 2.0 × 10^−1^ s^−1^. The effect of the addition of Ni into Sn-6.4Sb-3.9Ag on tensile strength is negligible at both 25 °C and 175 °C. The elongation of Sn-6.4Sb-3.9Ag decreases with an increase in the Ni addition volume at 25 °C according to the fracture mode change from ductile chisel point fracture to shear fracture. The effect of the addition of Ni into Sn-6.4Sb-3.9Ag on the elongation is negligible at 175 °C. The low cycle fatigue test result shows that the fatigue life does not degrade even at 175 °C in all alloys investigated. The fatigue life of Sn-6.4Sb-3.9Ag-0.4Ni (mass%) is superior to those of Sn-6.4Sb-3.9Ag and Sn-6.4Sb-3.9Ag-0.03Ni (mass%) in the high cycle fatigue area. The electron back scattering diffraction (EBSD) analysis result shows that fine recrystallized grains are generated at the cracked area in Sn-6.4Sb-3.9Ag-0.4Ni in the fatigue test at 175 °C, and the crack progresses in a complex manner at the grain boundaries.

## 1. Introduction

Since the restriction of hazardous substances (RoHS) directive was enforced in the European Union (EU) in 2006, the use of lead-containing solder in electrical and electric equipment has been regulated and thus lead-free solder such as Sn-Ag-Cu, Sn-Cu, and Sn-Bi alloys has been used [1]. Currently, Sn-Ag-Cu alloys such as Sn-3.0Ag-0.5Cu (mass%) and Sn-3.5Ag-0.7Cu (mass%) are mainly used for the assembly of electronics parts [2,3]. In the soldering process, solder is melted by heating, and molten solder wets the electrode of the electronics parts. After molten solder reacts with the electrode materials by the interdiffusion of constituent atoms, the joint is formed by the solidification of molten solder. Thus, many studies of the wettability of molten lead-free solder have been conducted [4,5,6]. Additionally, as the reliability of the soldered joint, the thermal fatigue resistance, the heat resistance and ion migration resistance are very important. Then, there are many studies for fatigue [7,8], thermal fatigue [9,10,11], interfacial reaction (growth of intermetallic compounds) [12,13,14], creep [15,16] and ion migration [17,18] of various lead-free solder. In particular, reliable thermal fatigue properties of the solder joint are among the most important factors under operating conditions. Moreover, finite element analysis of the electronics products is usually conducted to design the joint and predict its reliability. Various mechanical properties of the solder are required to conduct finite element analysis, and thus many studies on the mechanical properties of various lead-free solder have been conducted [19,20,21].

For the die attach material of the power semiconductor, lead-rich solder has been used due to its excellent thermal fatigue resistance. Although the lead-rich solder applied to some products is currently excluded from the RoHS directive, lead-free materials are expected to be applied to them in the future. In addition, Si has been used for the power semiconductor or SiC and GaN are expected to be next-generation semiconductors as such semiconductors can operate at a higher temperature and frequency, and with lower energy. In such next-generation semiconductors, operation temperature and surrounding environmental temperature are higher and thus the die attach materials must have improved thermal fatigue resistance. Therefore, various lead-free solders with higher solidus and liquidus temperatures than the Sn-Ag-Cu solder [22,23,24,25,26,27] and various nanopaste [28,29,30,31] have been researched to develop die attach materials with excellent thermal fatigue resistance.

We focused on Sn-Sb system alloys as high-temperature lead-free solder and investigated their mechanical properties and microstructures [24,25,26]. In particular, fatigue properties at temperatures ranging from 25 °C to 200 °C were investigated using micro-size specimens [8]. As a result, it was clarified that the addition of a small amount of Ni and Ge into the Sn-6.4Sb-3.9Ag (mass%) ternary peritectic-eutectic alloy can slightly improve the fatigue life of the alloy [26]. Moreover, it was already reported that the addition of a small amount of Ni and Ge into several lead-free solder improves the wettability of molten solder to the Cu plate [6]. In a previous study [26], the addition volume of Ge was very small (0.003 mass%) compared with that of Ni (0.25 mass%). Since the addition of Ge is effective to suppress the generation of the dross of solder and improve the wettability of the molten solder [32], it seems that the addition of Ni has a great effect on improving the mechanical properties of the alloy.

Therefore, the aim of this study is to investigate the effect of the addition volume of a small amount of Ni was investigated on microstructures, tensile properties and fatigue properties of Sn-6.4Sb-3.9Ag using micro-size specimens.

## 2. Materials and Methods

In this study, three kinds of Sn-Sb-Ag system lead-free solder were prepared. Table 1 shows their chemical compositions and melting properties. Melting properties were investigated by the differential scanning calorimetry method on the basis of JIS Z 3198-1 [33].

**Figure 1:**
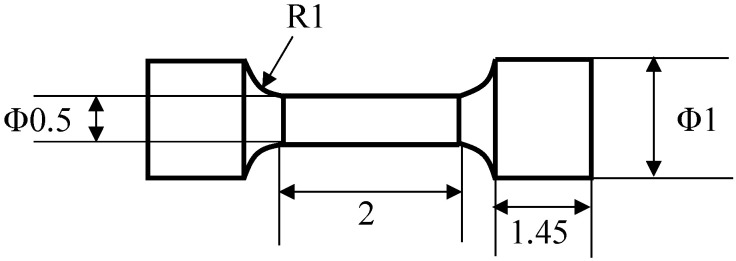
Shape and dimensions of micro-size specimen (Φ: diameter; unit: mm).

**Figure 2:**
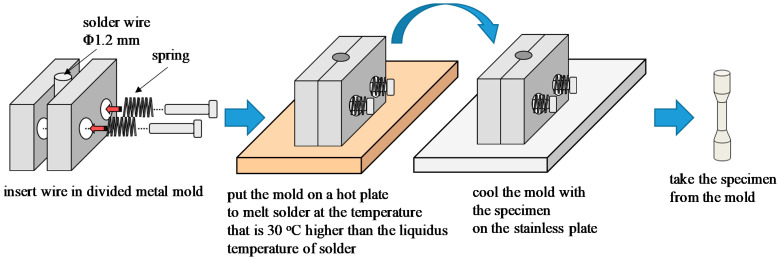
Fabrication flow of micro-size specimen.

**Figure 3:**
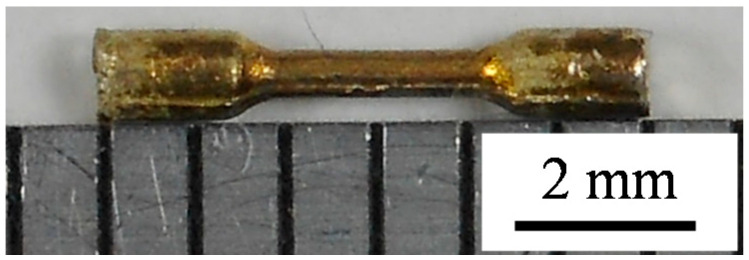
Appearance of micro-size specimen (Sn-6.4Sb-3.9Ag-0.4Ni).

Micro-size specimens were fabricated by Kariya’s method [8]. The shape and dimensions of the micro-size specimen are indicated in Figure 1. The fabrication flow of the specimen is shown in Figure 2. Solder wire with 1.2 mm diameter for each alloy was prepared. As-cast dog-bone type micro-size specimens were fabricated with solder wire using a divided mold. The gage length and the diameter of the specimen were 2 mm and 0.5 mm, respectively. Casting was conducted with a hot plate at a temperature which is 30 °C higher than the liquidus temperature of each solder. The cooling rate in the solidification process was controlled by removing the metal mold with the specimen from the hot plate and promptly cooling it on the stainless plate. The cooling rates of Sn-6.4Sb-3.9Ag, Sn-6.4Sb-3.9Ag-0.03Ni and Sn-6.4Sb-3.9Ag-0.4Ni in solidification were 3.0 °C/s, 2.2 °C/s and 2.5 °C/s, respectively. Figure 3 shows the appearance of the micro-size specimen.

A tensile test was carried out at a strain rate ranging from 2.0 × 10^−3^ s^−1^ to 2.0 × 10^−1^ s^−1^ at 25 °C and 175 °C using a microload test system (LMH207-10, SAGINOMIYA SEISAKUSHO, Inc., Tokyo, Japan). Five specimens were tested under each condition and tensile strength, 0.1% proof stress and elongation after fracture were investigated. Fractured specimens were observed with a field emission scanning electron microscope (FE-SEM) (S-4300SE, Hitachi High-Tech Science, Inc., Tokyo, Japan) after the tensile test. A low cycle fatigue test was also carried out by displacement control with the microload test system. The fatigue test was performed at a strain rate of 2.0 × 10^−3^ s^−1^ and temperatures of 25 °C and 175 °C. Displacement was controlled by a symmetrical triangle wave. The total strain range, Δε_t_ was controlled in the range of 0.5% to 2.5%. The fatigue life in this study was defined as a cycle number in which the maximum load was decreased by 20% compared with that at five cycles. To investigate the microstructures of the specimens before and after the fatigue test, the specimens were polished by #500–#4000 waterproof abrasive papers and subsequently polished with 1 μm alumina powder suspension. Thereafter, sputter etching by Ar milling was applied to the polished surfaces using a cross section milling machine (IB-19530CP, JEOL Ltd., Tokyo, Japan). An electron backscattering diffraction (EBSD) analysis was conducted on the specimen after Ar milling to investigate fatigue damage behavior using an EBSD system (TSL MSC-2200, TexSEM Laboratories, Inc., Provo, UT, USA) equipped with the FE-SEM. The rolling direction and the normal direction were set to the longitudinal direction of the specimen and the normal direction to the plane parallel to the longitudinal direction, respectively. In the EBSD analysis, Kikuchi lines of β-Sn were detected. The inverse pole figure (IPF) map and the grain boundary (GB) map were investigated. In this study, the grain boundary with a crystal orientation difference from 2° to 15° was defined as the low-angle grain boundary, and that with a crystal orientation difference from 15° to 180° was defined as the high-angle grain boundary. For the initial microstructure, the microstructural observation was also conducted with an electron probe X-ray microanalyzer (EPMA) (EPMA-1610, Shimadzu Corp., Kyoto, Japan).

## 3. Results and Discussion

### 3.1. Initial Microsturutures

**Figure 4:**
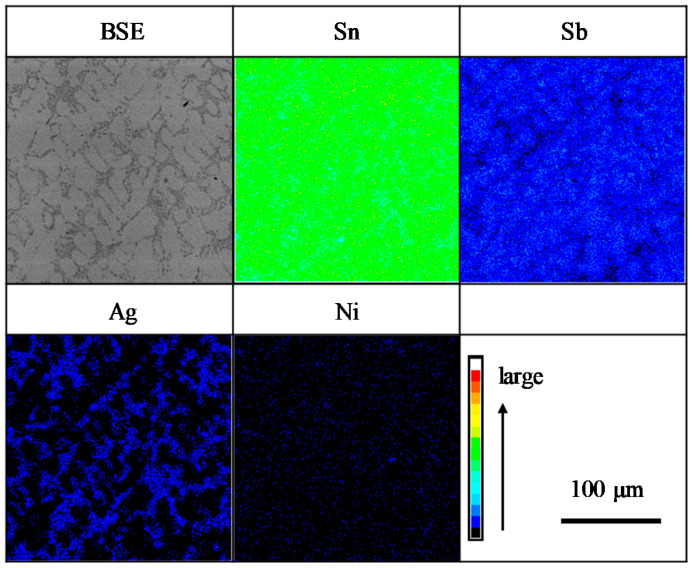
Result of mapping analysis for backscattered electron (BSE) image of as-cast Sn-6.4Sb-3.9Ag-0.4Ni micro-size specimen with electron probe X-ray microanalyzer (EPMA).

**Figure 5:**
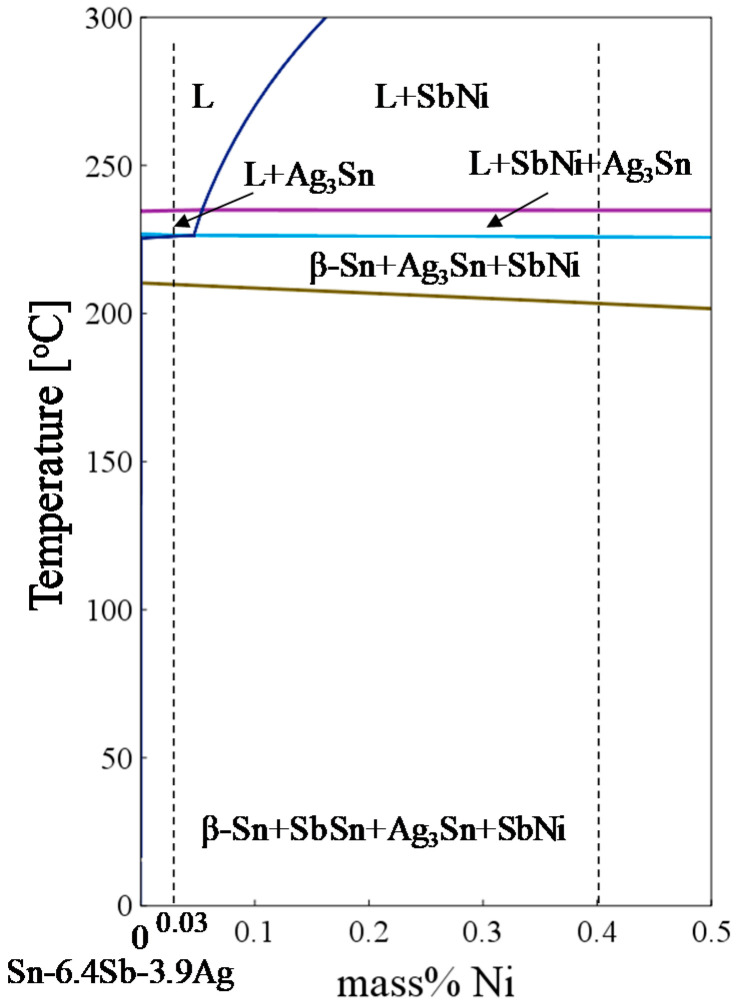
Phase diagram of Sn-6.4Sb-3.9Ag-xNi calculated by Thermo-Calc 2017a.

**Figure 6:**
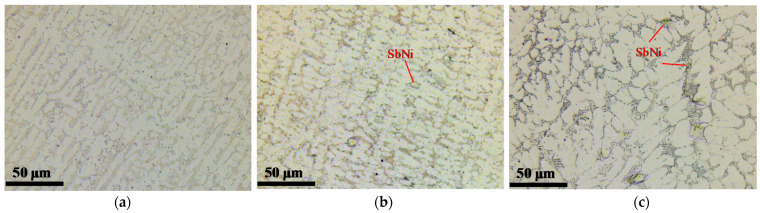
Optical microscope images of Sn-6.4Sb-3.9Ag (**a**), Sn-6.4Sb-3.9Ag-0.03Ni (**b**) and Sn-6.4Sb-3.9Ag-0.4Ni (**c**). The horizontal direction in the figure corresponds to the longitudinal direction of the specimen.

Figure 4 shows the result of the mapping analysis for the cross section of the as-cast Sn-6.4Sb-3.9Ag-0.4Ni micro-size specimen using the EPMA. The phase diagram of Sn-6.4Sb-3.9Ag-xNi calculated by Thermo-Calc 2017a (Thermo-Calc Software AB, Solna, Sweden) [34] is shown in Figure 5. In the backscattered electron (BSE) image, bright gray areas and dark gray areas are observed. Sn and Sb are detected in the bright gray area and Ag and Sn are detected in the dark gray area in Figure 4. Ni is condensed in some areas. Considering the EPMA mapping analysis and the phase diagram of Sn-6.4Sb-3.9Ag-xNi, SbSn, Ag_3_Sn and a small amount of SbNi seem to be dispersed in the β-Sn matrix. A similar microstructure was observed in the Sn-6.4Sb-3.9Ag-0.03Ni micro-size specimen although only a small amount of condensation of Ni was observed. Figure 6 shows optical microscope images of micro-size specimens. Similar micrographs to the BSE image shown in Figure 4 can be observed. It seems that the eutectic microstructure of β-Sn and Ag_3_Sn is formed to surround the β-Sn dendrite phases. Compared with the images of Sn-6.4Sb-3.9Ag and Sn-6.4Sb-3.9Ag-0.03Ni, the size of primary β-Sn phases is analogous, although a few inferred phases of SbNi are observed in the image of Sn-6.4Sb-3.9Ag-0.03Ni. In contrast, coarse primary β-Sn phases are formed in Sn-6.4Sb-3.9Ag-0.4Ni compared with other alloys. This means that the addition of 0.4%Ni promotes the coarsening primary β-Sn phases.

**Figure 7:**
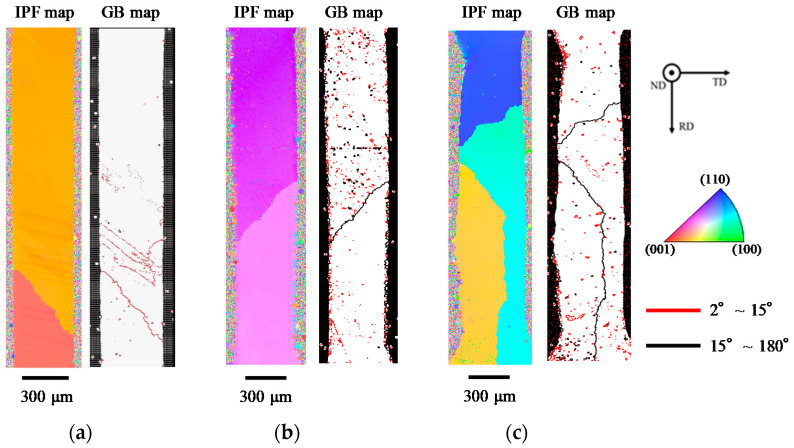
Electron backscattering diffraction (EBSD) analysis results of initial microstructures of Sn-6.4Sb-3.9Ag [26] (**a**), Sn-6.4Sb-3.9Ag-0.03Ni (**b**) and Sn-6.4Sb-3.9Ag-0.4Ni (**c**).

Figure 7 shows EBSD analysis results for as-cast microstructures of Sn-6.4Sb-3.9Ag [26], Sn-6.4Sb-3.9Ag-0.03Ni and Sn-6.4Sb-3.9Ag-0.4Ni alloys. In the micro-size specimens, there are one or, at most, a few grains in the cross section, which is perpendicular to the longitudinal direction [8]. In the Sn-6.4Sb-3.9Ag alloy, there are two grains with a low-angle grain boundary [26]. As shown in Figure 7b, although two grains are also observed in the Sn-6.4Sb-3.9Ag-0.03Ni alloy, the grain boundary changes to a high-angle grain boundary. Moreover, in the Sn-6.4Sb-3.9Ag-0.4Ni alloy shown in Figure 7c, three grains with high-angle grain boundaries are observed. From these results, it was found that the addition of Ni into Sn-6.4Sb-3.9Ag tends to increase the number of grains and form a high-angle grain boundary. This is due to the fact that Ni works as a nucleation site in the solidification process and increases the grain number.

### 3.2. Effect of Ni Addition on Tensile Properties of Sn-6.4Sb-3.9Ag

**Figure 8:**
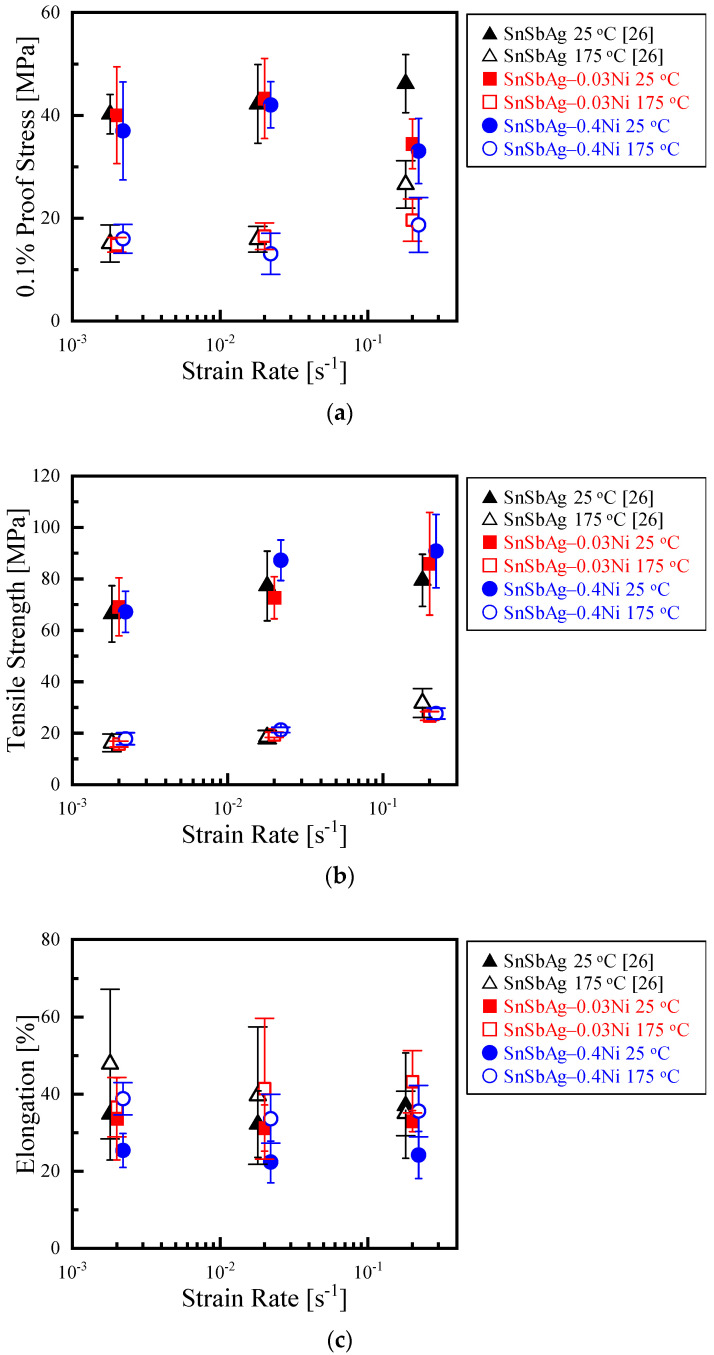
Tensile properties of Sn-6.4Sb-3.9Ag [26], Sn-6.4Sb-3.9Ag-0.03Ni and Sn-6.4Sb-3.9Ag-0.4Ni. (**a**) 0.1% proof stress; (**b**) tensile strength; (**c**) elongation.

Figure 8 shows the tensile properties investigated using micro-size specimens. The results of Sn-6.4Sb-3.9Ag are quoted from our previous study [26]. The 0.1% proof stress of Sn-6.4Sb-3.9Ag increases with an increasing strain rate at 25 °C. A similar tendency is found in the Sn-6.4Sb-3.9Ag with the addition of a small amount of Ni when the strain rate is less than 2.0 × 10^−2^ s^−1^. However, the 0.1% proof stress of the Sn-6.4Sb-3.9Ag decreases with the increase in the Ni addition volume at a strain rate of 2.0 × 10^−1^ s^−1^. As shown in Figure 7, the high-angle grain boundary is generated in the specimen by the addition of Ni. Then, the grains with various Schmidt factors form in the Ni containing Sn-6.4Sb-3.9Ag alloys. For example, in Figure 7b, the Schmidt factors of the upper grain and the lower grain were 0.40 and 0.15, respectively. The Schmidt factor of the upper grain was 0.1, and those of other grains were 0.35, as shown in Figure 7c. In the specimen in which a grain with a high Schmidt factor formed, plastic deformation easily took place. Thus, it is possible that the grain with a high Schmidt factor was easily deformed, and the proof stress decreased at a high strain rate in the Ni containing Sn-6.4Sb-3.9Ag alloys. At 175 °C, the 0.1% proof stress increased with an increase in strain rate in all alloys investigated, although the 0.1% proof stress at 175 °C was smaller than that at 25 °C.

As shown in Figure 8b, it was found that the tensile strength of Sn-6.4Sb-3.9Ag increases with an increase in the strain rate regardless of the temperature. The effect of the addition of Ni into Sn-6.4Sb-3.9Ag on the tensile strength is negligible. The tensile strength of all alloys at 175 °C was approximately one-third of those at 25 °C.

**Figure 9:**
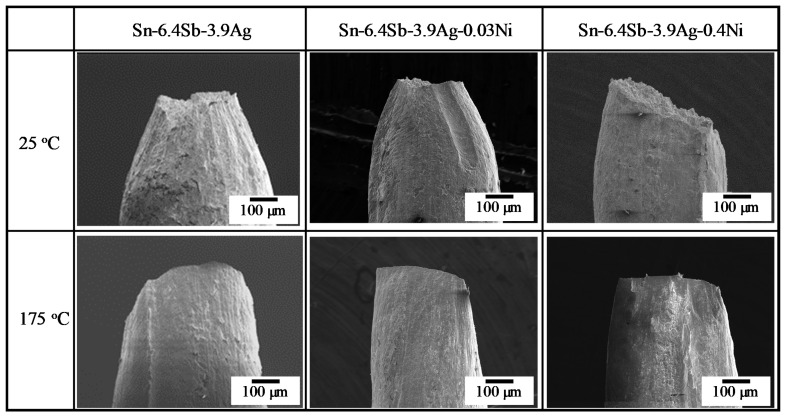
Secondary electron images of general views of ruptured specimens by tensile test at strain rate of 2.0 × 10^−1^ s^−1^.

For elongation, a clear relation was not seen between the strain rate and the elongation considering the large dispersion of the data. The elongation generally tended to improve with the increase in temperature, although the dispersion of the data was relative large. At 25 °C, the average of the elongation decreased with the increase in the Ni addition volume, although the decrease in Sn-6.4Sb-3.9Ag-0.03Ni was small. Figure 9 shows general views of ruptured specimens by the tensile test at a strain rate of 2.0 × 10^−1^ s^−1^. At 25 °C, the chisel point fracture was observed in Sn-6.4Sb-3.9Ag and Sn-6.4Sb-3.9Ag-0.03Ni. In contrast, shear fracture was observed in Sn-6.4Sb-3.9Ag-0.4Ni. This fracture mode change from ductile chisel point fracture to shear fracture caused a decrease in the elongation with the increase in the Ni addition volume. At 175 °C, a ductile chisel line fracture was observed, regardless of alloy types. Therefore, it seems that the meaningful differences were not observed in the elongation of the investigated alloys at 175 °C since the fracture mode did not change.

### 3.3. Effect of Ni Addition on Fatigue Properties of Sn-6.4Sb-3.9Ag

**Figure 10:**
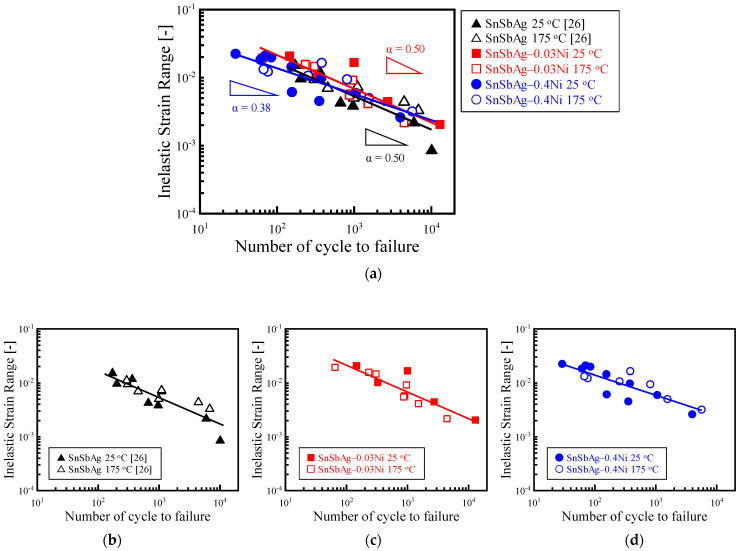
Fatigue properties of Sn-6.4Sb-3.9Ag [26], Sn-6.4Sb-3.9Ag-0.03Ni and Sn-6.4Sb-3.9Ag-0.4Ni. The results of all alloys are shown in (**a**), the results of Sn-6.4Sb-3.9Ag, Sn-6.4Sb-3.9Ag-0.03Ni and Sn-6.4Sb-3.9Ag-0.4Ni are shown in (**b**–**d**) respectively.

Figure 10 shows the relation of the inelastic strain range obtained from the hysteresis loop in the fatigue test and the number of cycle to failure obtained from the fatigue test. Since the obtained data were distributed relatively densely, the individual diagrams for each alloy are also shown in the figure. The result of Sn-6.4Sb-3.9Ag is quoted from our previous study [26]. In the low cycle fatigue test in which inelastic deformation becomes dominant, it is known that the relationship between the inelastic strain range and the number of cycles to failure follows the Manson–Coffin equation [35,36,37].

Δε_p_ = C_p_·(*N*_f_)^−α^ (1)

Δε_p_: inelastic strain amplitude, C_p_: fatigue ductility coefficient,

*N*_f_: fatigue life, α: fatigue ductility index

As shown in Figure 10, it was found that linear regression is possible in all Manson–Coffin plots, regardless of the test temperature. This means that the fatigue life does not degrade at 175 °C in all alloys investigated, and thus, they have excellent thermal fatigue resistance. The fatigue ductility indexes of Sn-6.4Sb-3.9Ag and Sn-6.4Sb-3.9Ag-0.03Ni were 0.50, and that of Sn-6.4Sb-3.9Ag-0.4Ni was 0.38. Since the Ni addition volume was a trace in the Sn-6.4Sb-3.9Ag-0.03Ni, the effect of the Ni addition on the fatigue property was negligible. In addition, the hypothesis that the fatigue life of Sn-6.4Sb-3.9Ag-0.4Ni is superior to those of other alloys in a high-cycle fatigue area was confirmed.

**Figure 11:**
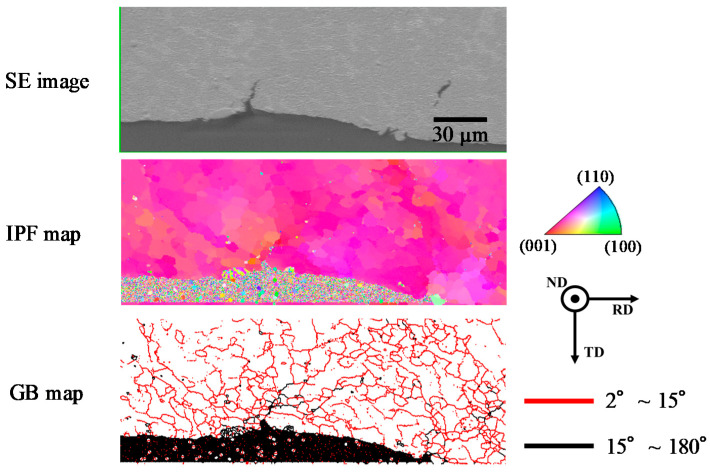
Result of EBSD analysis for fatigue crack area in Sn-6.4Sb-3.9Ag-0.4Ni formed by fatigue test at 25 °C (total strain amplitude: 1.0%, 1250 cycles, 20% load dropped).

The EBSD analysis result for the cracked area of Sn-6.4Sb-3.9Ag-0.4Ni formed by the fatigue test at 25 °C is shown in Figure 11. It was found that there are fine grains in the vicinity of the crack. As described later, in Sn with high stacking fault energy, fine grains were formed by continuous dynamic recrystallization with dynamic recovery in the thermal cycle test in the temperature range from −55 °C to 125 °C [38] and the fatigue test at 125 °C [23] and 200 °C [25]. The crack progresses at the grain boundaries of fine grains with high-angle grain boundaries [23,38]. As shown in Figure 11, it was found that the grains with high-angle grain boundaries scarcely formed in the fatigue test at 25 °C. Most of the grains observed in Figure 11 have low-angle grain boundaries. These grains seem to be sub-grains formed by dynamic recovery. At 25 °C, the recrystallization of β-Sn grains by the rotation of sub-grains scarcely occurred due to the low temperature, thus making the formation of the grains with high-angle grain boundaries difficult. As a result, the crack progressed at the grain boundaries of fine grains with low-angle grain boundaries. An analogous analysis result was also observed in Sn-6.4Sb-3.9Ag [26] and Sn-6.4Sb-3.9Ag-0.03Ni. There were almost no changes in the effect of the addition of Ni on the fatigue properties of Sn-6.4Sb-3.9Ag at 25 °C.

**Figure 12:**
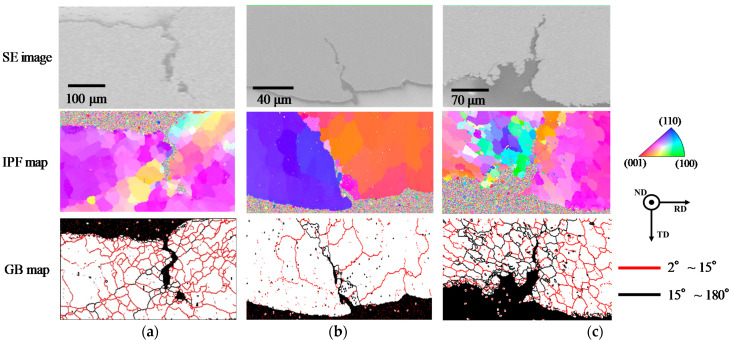
Result of EBSD analysis for fatigue crack areas formed by fatigue test at 175 °C. (**a**) Sn-6.4Sb-3.9Ag (total strain amplitude: 1.0%, 1009 cycles, 28% load dropped); (**b**) Sn-6.4Sb-3.9Ag-0.03Ni (total strain amplitude: 1.0%, 1127 cycles, 22% load dropped); (**c**) Sn-6.4Sb-3.9Ag-0.4Ni (total strain amplitude: 1.0%, 1057 cycles, 27% load dropped).

The EBSD analysis result for the cracked area of each alloy formed by the fatigue test at 175 °C is shown in Figure 12. As shown in Figure 12a, it was found that many fine recrystallized grains were generated in the vicinity of the crack. These fine grains were formed by continuous dynamic recrystallization [23,38]. Generally, in the metals such as Sn, which have high stacking fault energy, it was reported that the crack progressed at the grain boundaries of fine grains, which were generated by continuous dynamic recrystallization with dynamic recovery [23,38]. A similar phenomenon was observed in the fatigue damage behavior of Sn-6.4Sb-3.9Ag at 175 °C. Analogous fine recrystallized grain formation was also observed in Sn-6.4Sb-3.9Ag-0.4Ni, as shown in Figure 12c. Compared with Sn-6.4Sb-3.9Ag, relatively finer recrystallized grains were generated, and the number of grains with high-angle grain boundaries was large in Sn-6.4Sb-3.9Ag-0.4Ni. As described in Section 3.1, the addition of Ni into Sn-Sb alloys causes the formation of SbNi phases in the matrix [24,25,26]. Such SbNi phases became the origin of recrystallization, and continuous dynamic recrystallization with dynamic recovery occurred easily in Sn-6.4Sb-3.9Ag-0.4Ni. Thus, strain energy was consumed by continuous dynamic recrystallization, and strain energy used by the progress of the crack reduced. As a result, the progress of the crack was slightly restrained. In addition, the crack progressed in a complex manner at the grain boundaries of fine grains, and thus the progress of the crack was also restrained. Therefore, the fatigue life of Sn-6.4Sb-3.9Ag-0.4Ni was slightly improved at 175 °C.

**Figure 13:**
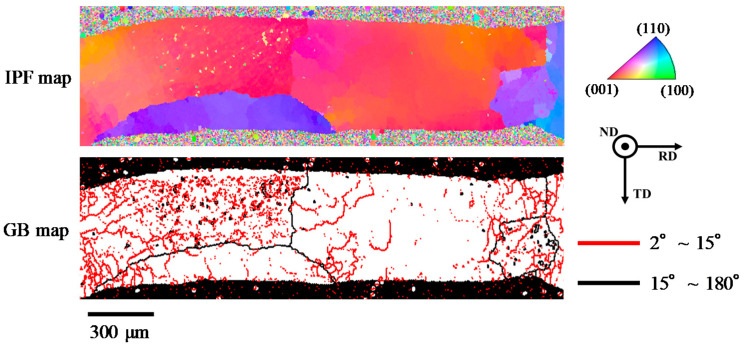
Inverse pole figure (IPF) and grain boundary (GB) maps of the whole specimen of Sn-6.4Sb-3.9Ag-0.03Ni, as shown in Figure 12b.

In contrast, fine grains formation was scarcely observed in the vicinity of the crack progress area in Sn-6.4Sb-3.9Ag-0.03Ni, as shown in Figure 12b. The crack progressed at the original high-angle grain boundary, and the grain boundary of fine recrystallized grains was generated in the vicinity of the crack. Figure 13 shows the IPF and GB maps of the whole specimen of Sn-6.4Sb-3.9Ag-0.03Ni, as shown in Figure 12b. Many low-angle grain boundaries were observed, except the original high-angle grain boundaries. As described in Section 3.1, the microstructure of Sn-6.4Sb-3.9Ag-0.03Ni is similar to that of Sn-6.4Sb-3.9Ag, although a few SbNi phases and a few high-angle grain boundaries exist in the microstructure. Thus, if the crack is not generated at the original high-angle grain boundary, the crack would generate at the grain boundary of fine grains as well as Sn-6.4Sb-3.9Ag, as shown in Figure 12a. Whether the crack generates at the original high-angle grain boundary or at the grain boundary of fine grains depends on the grain number and the orientations of the grains. In addition, it seems that the fatigue life was scarcely changed in both crack generation modes. Therefore, the fatigue properties of Sn-6.4Sb-3.9Ag-0.03Ni are almost equal to those of Sn-6.4Sb-3.9Ag. However, Figure 12b shows only the result of the crack that progressed at the original high-angle grain boundary and the grain boundary of fine recrystallized grains generated in its vicinity; therefore, further study is required to clarify the progress mechanism of the crack in Sn-6.4Sb-3.9Ag-0.03Ni.

## 4. Conclusions

The effect of the addition volume of a small amount of Ni on the microstructures was investigated, as was that on the tensile and fatigue properties of Sn-6.4Sb-3.9Ag, using micro-size specimens. The results obtained are summarized as follows.

(1) The addition of Ni into Sn-6.4Sb-3.9Ag tends to increase the number of grains formed in the solidification process and form a high-angle grain boundary.

(2) The 0.1% proof stress of Sn-6.4Sb-3.9Ag, Sn-6.4Sb-3.9Ag-0.03Ni and Sn-6.4Sb-3.9Ag-0.4Ni increases with an increase in strain rate at 25 °C and 175 °C when the strain rate is less than 2.0 × 10^−2^ s^−1^. The 0.1% proof stress of Sn-6.4Sb-3.9Ag decreases with an increase in the Ni addition volume at a strain rate of 2.0 × 10^−1^ s^−1^. The effect of the Ni addition into Sn-6.4Sb-3.9Ag on the tensile strength is negligible at 25 °C and 175 °C. The elongation of Sn-6.4Sb-3.9Ag decreases with an increase in the Ni addition volume at 25 °C according to the change in the fracture mode from ductile chisel point fracture to shear fracture. The effect of the addition of Ni into Sn-6.4Sb-3.9Ag on the elongation is negligible at 175 °C.

(3) The relation of the inelastic strain range and the cycle to failure obtained from the low cycle fatigue test in each alloy follows the Manson–Coffin equation. The result shows that the fatigue life does not degrade at 175 °C in all alloys investigated, and they have excellent thermal fatigue resistance. The fatigue life of Sn-6.4Sb-3.9Ag-0.4Ni is superior to those of Sn-6.4Sb-3.9Ag and Sn-6.4Sb-3.9Ag-0.03Ni in a high-cycle fatigue area. On the basis of the EBSD analysis result for the cracked area of Sn-6.4Sb-3.9Ag-0.4Ni formed by the fatigue test at 175 °C, it was found that finer recrystallized grains are generated at the cracked area by continuous dynamic recrystallization with dynamic recovery, and the crack progresses in a complex manner at the grain boundaries of fine grains, and thus the progress of the crack is restrained.

## Figures and Tables

**Table 1:** Chemical compositions and melting properties of alloys used in this study.

Chemical Composition [Mass%]	Solidus Temperature [°C]	Liquidus Temperature [°C]
Sn-6.4Sb-3.9Ag	230.4	234.8
Sn-6.4Sb-3.9Ag-0.03Ni	225.3	234.3
Sn-6.4Sb-3.9Ag-0.4Ni	226.3	233.8

## Data Availability

Data are contained within the article.
